# Perceptions Regarding Healthy Eating Based on Concept Mapping

**DOI:** 10.3390/nu17182941

**Published:** 2025-09-12

**Authors:** Gaeun Yeo, Jieun Oh

**Affiliations:** 1 Department of Nutritional Science and Food Management, Ewha Womans University, Seoul 03760, Republic of Korea; gaeunyeo@naver.com; 2 College of Science & Industry Convergence, Ewha Womans University, Seoul 03760, Republic of Korea

**Keywords:** healthy eating, eating perceptions, concept mapping method, eating lifestyles, eating context, food, food choice

## Abstract

**Background/Objectives**: This study aimed to examine contemporary perceptions of healthy eating among individuals living in a rapidly evolving social, economic, and cultural environment. A mixed-methods approach was employed using the concept mapping method to collect, visualize, and analyze participants’ perceptions. **Methods**: Twenty-four Korean individuals aged 16 to 55 participated in the study. Initially, perceptions of healthy eating were gathered through focus group interviews. These responses were organized into sixty-three unique statements. Participants were then asked to sort the statements and rate each one’s importance and performance on a 5-point Likert scale. The sorted and rated data were analyzed using R-CMap, an open-source software for concept mapping. **Results**: The analysis revealed six key clusters of healthy eating perceptions among Korean consumers: Food Choice, Nutrition, Eating Habits, Eating Environment, Production, and Preparation and Cooking. These perceptions are influenced by Korean food culture and the current eating context. In addition, a detailed analysis of the statements revealed that public perceptions of healthy eating have shifted in recent years. **Conclusions**: This research is significant in that it offers a structured and visualized framework of healthy eating perceptions among Koreans aged 16–55, reflecting their adaptation to ongoing socio-cultural transformations.

## 1. Introduction

Eating is a concept that entails behaviors, norms, and attitudes related to human food intake, and it is deeply influenced by social, economic, and cultural contexts [1]. In the past, meals were a means of survival to appease hunger and supply energy to humans; however, they presently function as a cultural tool that reflects people’s values and preferences [2,3]. Accordingly, the concept of “eating” is continuously evolving. Its meaning varies with historical context, social norms, cultural background, individual values, preparation methods, convenience, food functions, and health awareness [3,4].

Among these factors, health has long been a major motivation and factor in food choice. Therefore, several studies have investigated the concept of healthy eating. To date, the traditional view of healthy eating has been dominated by food science, which conceptualizes eating as nutrient acquisition and emphasizes sensory properties such as taste, smell, and texture [5,6]. However, over time, the scope of perceptions regarding eating has gradually expanded. In addition to nutrients and energy content, it also encompasses meal components, quantity, dietary guidelines, ethnic and cultural perspectives, food culture and personal identities [4,5,7]. Further, eating perceptions are considerably diverse and exhibit differences based on the research group’s characteristics, such as sex and age [7,8].

Meanwhile, Korean society has recently undergone significant structural changes, including increases in dual-income and single-person households as well as an aging population [9]. Also, technological innovations in food processing, delivery, and distribution systems have reshaped consumer choices, driving the industry to prioritize efficiency [8,10]. In addition, social media platforms such as Instagram and TikTok have influenced food behavior and trends, viewing eating from gastronomic and leisure perspectives [11].

Furthermore, the COVID-19 pandemic has accelerated the integration of various food technologies into daily life, leading to notable changes in eating habits and consumption patterns [12]. As more people ate at home, grocery and food delivery expenses rose dramatically and demand for delivery and processed foods significantly increased [9,13,14]. These changes have reshaped consumer behavior and expectations regarding food choices. In response, the food industry has upgraded the development and production of convenience food products, restaurants have realized a surge in delivery and takeout sales, and online ordering platforms have become more popular, all of which have altered the market along with consumer behavior and habits [12,15]. In addition, COVID-19 significantly heightened people’s health concerns, leading to greater demand for healthy eating due to activity restrictions such as social distancing and quarantine [14,16,17,18]. However, despite this increased demand, some studies have reported that lifestyle changes and psychological stress during the pandemic have altered people’s food choices, resulting in less healthy and more irregular eating patterns [16,18,19,20,21].

Collectively, these socio-environmental changes and developments have led to significant shifts in people’s eating lifestyles, which in turn directly influence their perceptions and values regarding food. However, despite these shifts, there is still limited research specifically examining how consumers currently perceive healthy eating within this rapidly changing socio-cultural environment. Given these circumstances, concept mapping provides a particularly suitable methodological approach for exploring how consumers define healthy eating. This method not only captures the multidimensional and collective nature of perceptions but also integrates cognitive, emotional, and experiential aspects into a structured framework [22]. Such strengths make concept mapping uniquely valuable in the Korean sociocultural context, where rapid transformations in technology, family structure, and food-related practices require a comprehensive tool to reveal both cultural continuity and change.

Therefore, the present study applies the concept mapping method to investigate how Korean consumers perceive healthy eating in the current context of rapid socioeconomic and cultural change.

In addition, recognizing that groups with higher socioeconomic and educational status are socially influential and tend to lead lifestyle trends [23], this study recruited participants residing in the Seoul metropolitan area with above-average socioeconomic and educational status. This focus was intended to capture the most influential and current food perceptions in contemporary Korean society.

## 2. Materials and Methods

### 2.1. Study Design

This study employed a concept mapping approach to examine contemporary perceptions of healthy eating among Korean adults. The concept mapping method, developed by Trochim WM [22], is designed to collect and structure individual perceptions and opinions, presenting the results visually as a map. This mixed-methods approach integrates both qualitative and quantitative techniques to systematically organize the internalized thoughts or perceptions of a group into a coherent conceptual framework [24,25]. Concept mapping is particularly well-suited for exploring perceptions and concepts that lack a well-established theoretical foundation, effectively revealing the components and structure of a given research topic by incorporating participants’ experiences and perspectives.

**Figure 1:**
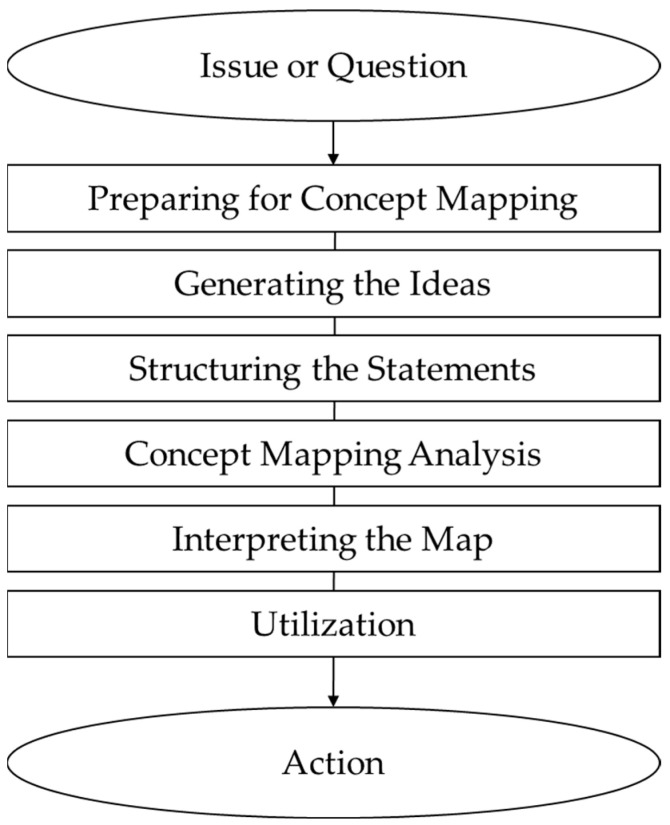
Concept mapping process.

Following this approach, a concept mapping study typically involves six stages (Figure 1): (1) preparation; (2) generation of statements; (3) structuring of statements; (4) representation of statements; (5) interpretation of maps; (6) utilization of maps. The process begins with the development of a focus question and the collection of ideas from participants, which are synthesized into concise statements that reflect key themes. Participants then sort and rate these statements, providing data for constructing the conceptual framework. Although grounded in qualitative methodology, this approach also incorporates quantitative elements, enabling a detailed exploration of participants’ experiences and subjective perceptions while producing structured, interpretable results rather than a simple list of disparate ideas [26].

Concept mapping studies have been actively applied in counseling psychology and education, and their use is expanding into fields such as healthcare, medicine, public health, and policy. For example, Vizireanu M and Hruschka D [27] explored perceptions of healthy eating in the United States, categorizing them along dimensions such as food characteristics, eating style, and trusted advice. Rauwerdink et al. [28] used concept mapping to develop a strategic vision for an eHealth program at a Dutch university medical center. Svobodova et al. [29] identified strategies for improving healthcare access among the Roma population, while McNeil et al. [30] examined innovative local health systems for seniors and caregivers in Canada. Vaingankar et al. [31] conducted a qualitative concept mapping study on the benefits of social media for children’s mental health in Singapore. In addition, Tsui et al. [32] utilized concept mapping methods to establish strategies for HPV vaccination, demonstrating its relevance in current public health planning.

In the Korean context, this method is also appropriate for systematically analyzing consumers’ unstructured and diverse perceptions of healthy eating, which are shaped by cultural, social, and technological influences. As such, concept mapping offers an effective approach for systematically analyzing unstructured ideas in diverse fields while supporting practical, culturally relevant applications in research.

The present study was conducted in two phases, aligning with the dual qualitative-quantitative nature of concept mapping. First, qualitative data were collected via focus group interviews to generate statements on healthy eating perceptions among Koreans. Subsequently, a quantitative study was conducted in which the extracted statements were sorted and rated to identify the structural components and relative importance of these perceptions. The study protocol was approved by the Institutional Review Board of Ewha Womans University, Seoul, Republic of Korea (IRB No. EWHA-202210-0002-01).

### 2.2. Participants

Participants were recruited using a snowball sampling method. In this study, participants were limited to Korean individuals aged 16 to 55 who had lived in the Republic of Korea for at least 10 years, possessed up-to-date knowledge of Korean food culture, resided in the Seoul metropolitan area, and had above-average socioeconomic and educational status, which was intended to ensure familiarity with diverse dietary environments and informed reflections on eating behaviors. These higher-educated, higher-SES individuals are often regarded as possessing greater cultural capital and exerting significant influence on shaping lifestyle and food perceptions [23,33]. Furthermore, education has been shown to affect food purchasing through dietary knowledge, while income shapes choices through food-cost concerns [34]. Thus, focusing on this group allowed the study to capture influential perspectives on healthy eating in contemporary Korean society. Individuals with academic or professional backgrounds in food-related fields were excluded.

Although concept mapping does not require a strict number of participants, Trochim W and Kane M [25] recommend 8–40 participants, and several studies suggest that approximately 20 participants are sufficient [29,35,36]. Based on these guidelines and the study’s objectives, 24 Korean participants were selected. The age distribution included 4 participants in their teens, 5 in their 20s, 6 in their 30s, 6 in their 40s, and 3 in their 50s. Gender was evenly distributed, with 12 male and 12 female participants. Teenagers were defined as individuals aged 16 or older, as this age group is generally capable of independently purchasing and consuming food. Participation for minors included documented informed consent from legal guardians and an assent process with the participants themselves. To ensure continuity and deeper engagement with the research process, individuals who participated in the initial qualitative phase (interviews) were also invited to participate in the subsequent structuring phase (statement sorting and rating) [24].

### 2.3. Part 1: Generation of Healthy Eating Perceptions Statements

#### 2.3.1. Procedure of Focus Group Interview

To conduct this study, we developed (1) a focus question (“*What do you perceive as healthy eating*?”) to initiate the overall interview, which involved themes generated by brainstorming and reviewing previous studies and literature [24] and (2) a facilitating question (*“When you eat, do you generally feel that you are eating healthily or believe that your eating habits are healthy? Based on your personal experiences or values, how would you define ‘healthy eating’ ?”*) to elicit various responses from the participants.

Focus group interviews were conducted in October 2022 and administered online. The researcher briefed the participants online regarding the purpose of the study and interview and subsequently presented the focus questions on the screen. Thereafter, participants were asked to take turns expressing their perceptions regarding healthy eating by recalling their own experiences and major thoughts. A total of six focus-group-interview sessions, each lasting 60–120 min, were conducted.

#### 2.3.2. Focus Group Interview Transcripts Analysis

After the interviews were completed, they were transcribed. First, the content mentioned by the research participants was processed into basic statements, from which 488 semantic unit sentences were derived. These semantic unit sentences were subjected to a keyword extraction process, and the sentences were rearranged based on the similarity of extracted keywords with the assistance of three experts in the food field. Similar concepts were combined into single statements, while overlapping concepts were separated or reworded to clearly represent the intended meaning. Statements mentioned by fewer than two participants were excluded [37].

All statements were then reformulated in the structure: “Healthy eating is *[statement]*.” A total of 82 statements were generated. Next, these statements were evaluated for relevance to the concept of “healthy eating perception.” Five experts with doctoral degrees and expertise in food and nutrition were provided with an evaluation questionnaire using a 5-point Likert scale, along with a comment sheet. Statements that received a score of less than 3 from all five experts were excluded from further analysis. The remaining statements were revised based on the expert feedback. Through this iterative review, which was based on expert evaluation and revisions informed by feedback and consensus, the final set of 63 statements was established, thereby strengthening the content validity of the instrument.

### 2.4. Part 2: Structuring Statements of Healthy Eating Perceptions

The statement-structuring task involved sorting and rating 63 statements and was completed by 24 participants. Each participant received a research packet containing an instruction sheet, 63 statement cards (7.6 × 12.7 cm), a printed list of statements, a similarity-sorting sheet, “Importance” and “Performance” rating sheets, and a paperclip. Participants completed the task individually and submitted their results by email (scanned forms) to the researcher.

For the sorting task, participants were asked to spread out the statement cards, read through them, and group them by similarity. They were then asked to bundle each group using the provided paperclip. The sorting procedure followed the guidelines of Kane M and Trochim WM [24], which include: (1) all statement cards must be grouped; (2) no card may be left ungrouped; (3) each group must contain at least two cards; (4) a statement may not belong to more than one group; and (5) no group may include more than one-third of the total number of statements.

Participants recorded the statements contained in each group on the similarity-sorting sheet and were asked to assign a label to each group that best reflected its underlying concept. Following this, they rated each of the 63 statements for both Importance and Performance ratings using separate 5-point Likert scales. For importance, ratings ranged from 1 (“not at all important”) to 5 (“very important”), based on how important each statement was for ‘healthy eating.’ For performance, participants rated the extent to which they practiced each behavior, from 1 (“not at all”) to 5 (“a lot”).

The task took approximately 60 to 120 min to complete, and data collection was conducted in November 2022.

### 2.5. Data Analysis

Before conducting the analysis, we coded the statement sorting and rating data provided by the participants. The analysis was carried out using R-CMap (https://haim-bar.uconn.edu/software/r-cmap/, accessed on 27 August 2025) an open-source software program [38].

Initially, a two-dimensional MDS (multidimensional scaling) analysis was performed using the sorting data. In MDS analysis, it is essential to determine an appropriate number of dimensions for the study. When combined with cluster analysis, a two-dimensional solution is generally more practical and interpretable than models with three or more dimensions [39]. In concept mapping studies, it is also common to represent statements on a two-dimensional map using MDS [24].

Next, the resulting coordinates from the MDS analysis were used to conduct hierarchical cluster analysis using the complete linkage method. Each statement was grouped into clusters based on similarity. These clusters, along with the participants’ rating data, were subsequently used to generate and analyze visual representations such as Go-Zone graphs and pattern-matching graphs.

In addition, paired t-tests were conducted using SPSS 26.0 (IBM Corp, Armonk, NY, USA) to examine whether the mean differences between importance and performance were statistically significant within each cluster.

## 3. Results and Discussion

### 3.1. Clustering of the Healthy Eating Perceptions

**Figure 2:**
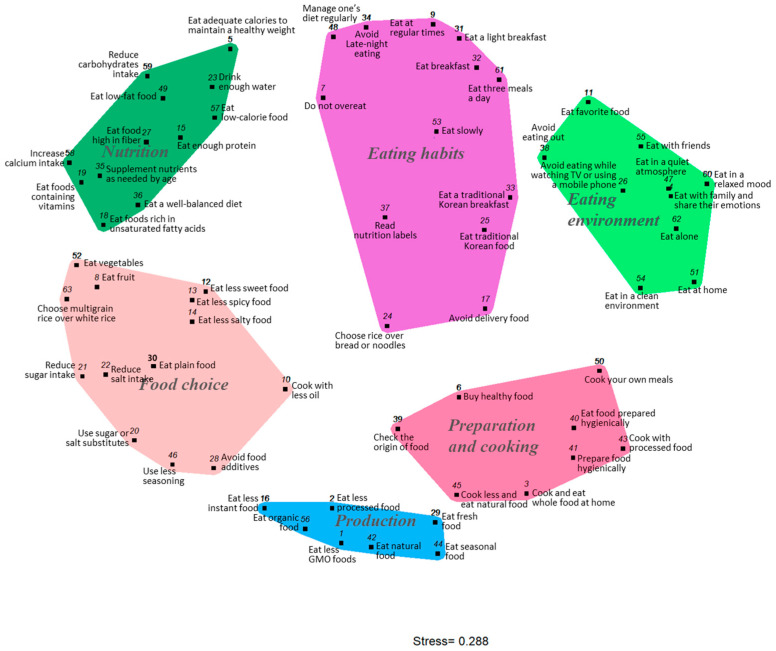
Cluster map of the healthy eating perceptions.

Based on the sorted data on perceptions of healthy eating, we conducted a multidimensional scaling (MDS) analysis and presented the results as a point map (Figure 2). The 63 statements were positioned within a two-dimensional space according to how participants sorted them, so that statements with higher perceived similarity were placed closer together, while those with lower similarity were positioned farther apart. To evaluate the goodness of fit for the MDS analysis, the stress value is commonly examined. This value reflects the degree of mismatch between distances on the MDS map and the actual similarity data; the lower the stress value, the better the fit. In this study, the stress value was 0.288, which is within the acceptable range (0.205–0.365) reported in previous studies [22,24].

Each statement was assigned to a cluster through hierarchical cluster analysis (Figure 2). In concept mapping research, the number of clusters is not predetermined but is typically determined by the researcher’s judgment. It is generally recommended that the researcher specify the maximum and minimum number of clusters and then examine each solution sequentially to identify the most appropriate cluster structure [24]. In this study, three experts were consulted to assess the appropriateness of the statement groupings and cluster classifications, resulting in a final solution of six clusters, each containing between 6 and 14 statements.

Cluster 1 includes the following statements: “*8. Eat fruit*,” “*10. Cook with less oil*,” “*12. Eat less sweet food*,” “*13. Eat less spicy food*,” “*14. Eat less salty food*,” “*20. Use sugar or salt substitutes*,” “*21. Reduce sugar intake*,” “*22. Reduce salt intake*,” “*28. Avoid food additives*,” “*30. Eat plain food*,” “*46. Use less seasoning*,” “*52. Eat vegetables*,” and “*63. Choose multigrain rice over white rice*.” The statements in this cluster primarily relate to specific foods and ingredients considered appropriate for a healthy diet. Ishak et al. [40] categorized fruit and vegetable consumption under “Food types and characteristics,” Vizireanu M and Hruschka D [27] classified low-sugar and low-salt preferences as food characteristics, and Kaya IH [41] discussed flavor preferences in the context of food choice. Thus, this cluster reflects specific food and ingredient preferences and was named *Food Choice.*

Cluster 2 comprises the following statements: “*5. Eat adequate calories to maintain a healthy weight*,” “*15. Eat enough protein*,” “*18. Eat foods rich in unsaturated fatty acids*,” “*19. Eat foods containing vitamins*,” “*23. Drink enough water*,” “*27. Eat foods high in fiber*,” “*35. Supplement nutrients as needed by age*,” “*36. Eat a well-balanced diet*,” “*49. Eat low-fat food*,” “*57. Eat low-calorie food*,” “*58. Increase calcium intake*,” “*59. Reduce carbohydrates intake*,” along with 12 additional statements. These items primarily address essential nutrient intake and dietary balance. Therefore, the cluster was labeled Nutrition, drawing on the categorization by Chen et al. [42], who identified the intake of fat, calories, fiber, sugar, and salt as indicators of nutritional value.

Cluster 3 consisted of 13 statements: “*7. Do not overeat*,” “*9. Eat at regular times*,” “*17. Avoid delivery food*,” “*24. Choose rice over bread or noodles*,” “*25. Eat traditional Korean food*,” “*31. Eat a light breakfast*,” “*32. Eat breakfast*,” “*33. Eat a traditional Korean breakfast*,” “*34. Avoid late-night eating*,” “*37. Read nutrition labels*,” “*48. Manage one’s diet regularly*,” “*53. Eat slowly*,” and “*61. Eat three meals a day*.” These statements mainly relate to eating patterns—such as breakfast consumption, meal regularity, and portion control—as well as behavioral habits including nutrition label use and regular diet management. Accordingly, the cluster was designated *Eating Habits*, referencing the classifications proposed by Voytyuk M and Hruschka D [7] and Ishak et al. [40], who define eating habits to encompass behaviors like avoiding overeating and eating slowly.

Cluster 4 consisted of 10 statements: “*4. Eat with family and share emotions*,” “*11. Eat favorite food*,” “*26. Avoid eating while watching TV or using a mobile phone*,” “*38. Avoid eating out*,” “*47. Eat in a quiet atmosphere*,” “*51. Eat at home*,” “*54. Eat in a clean environment*,” “*55. Eat with friends,” “60. Eat in a relaxed mood*,” and “*62. Eat alone*.” These statements emphasize the physical and social context in which eating occurs, including dining companions, setting, and emotional atmosphere. In the study by Haerens et al. [43], behaviors such as eating while watching television and maintaining healthy eating routines at home were classified as home environment factors. Given these features, the cluster was titled *Eating Environment*.

Cluster 5 contains seven statements: “*1. Eat less GMO food*,” “*2. Eat less processed food*,” “*16. Eat less instant food*,” “*29. Eat fresh food*,” “*42. Eat natural food*,” “*44. Eat seasonal food*,” and “*56. Eat organic food*.” These items are primarily concerned with food production methods and origins, including preferences for natural, organic, and minimally processed products. In the Food Information Literacy Scale developed by the National Academy of Agricultural Sciences [44], questions related to genetically modified foods, agri-food certification, and processed food consumption are categorized under the domain of production. This grouping emphasizes production and origin aspects and was categorized as *Production*.

Cluster 6 consists of eight statements: “*3. Cook and eat whole food at home*,” “*6. Buy healthy food*,” “*39. Check the origin of food*,” “*40. Eat food prepared hygienically*,” “*41. Prepare food hygienically*,” “*43. Cook with processed food*,” “*45. Cook less and eat natural food*,” and “*50. Cook your own meals*.” These statements focus on the processes of food selection, handling, and preparation, including checking food origin, ensuring hygiene, and cooking at home. In the study by Park et al. [45], actions such as verifying hygiene and healthy food information during meal preparation were categorized under preparation and cooking factors. Based on this classification, the cluster was identified as *Preparation and Cooking*.

Korean consumers perceive healthy eating across six categories: Food Choice, Nutrition, Eating Habits, Eating Environment, Production and Preparation and Cooking. These perceptions are shaped by both Korean food culture and the current eating environment, reflecting changes such as increased eating out, delivery use, and evolving family meal pattens in Korean society.

Previous studies on healthy eating perceptions have primarily focused on food and nutrient intake [5,6,46]. More recent research has continued to emphasize the importance of food and nutrient intake in shaping perceptions of healthy eating [7,8,27]. Similarly, in this study, aspects of food selection (Food Choice cluster), ingredient intake (Nutrition cluster), and food-related perspectives (Production cluster) were identified. The Food Choice cluster includes statements related to the types and quantities of food that should be consumed for a healthy diet. The Nutrition cluster focuses on the types and methods of nutrient intake. The Production cluster contains statements regarding the origin, production methods, and manufacturing processes of food. The results of this study align with the findings reported by Chen et al. [42] and Lusk JL [47].

In addition, an Eating Habits cluster was identified, which regards healthy eating as a matter of eating habits, and an Eating Environment cluster, which frames healthy eating in terms of context and lifestyle. This tendency can be attributed to cultural characteristics [7]. In a study by Akamatsu et al. [48], Japanese participants tended to perceive healthy eating differently from Western cultures, viewing it not only as a nutritional aspect but also as an element of eating style and habits. Similarly, in the study by Voytyuk M and Hruschka D [7], Ukrainians were more likely than Americans to consider the context of eating, such as the time of day and eating style, when perceiving healthy eating. This difference can be explained by cultural factors, as eating perceptions often differ based on whether the culture tends to emphasize a collectivistic or individualistic approach.

Another cluster, called the preparation and cooking cluster, emerged that views healthy eating in terms of food preparation and handling. Consistent with previous studies [49,50], which emphasize the importance of food preparation and cooking skills for healthy eating, we found that participants also perceive the preparation and cooking of food as essential components of healthy eating.

### 3.2. Importance and Performance of Healthy Eating Clusters

Participants’ ratings of the importance and performance of healthy eating were analyzed at the cluster level (Table 1).

The first cluster, Food Choice, consisted of 13 statements with an average importance rating of 3.95 and a performance rating of 3.22. This cluster reflected perceptions of healthy eating in terms of selecting and consuming appropriate foods. The second cluster, Nutrition, included 12 statements, with the highest average importance score (3.99) but a relatively lower performance score (3.08). This cluster captured perceptions centered on nutrient intake and dietary composition. The third cluster, Eating Habits, comprised 13 statements, with average ratings of 3.62 for importance and 3.03 for performance. The statements reflected individual habits and beliefs concerning healthy eating practices. The fourth cluster, Eating Environment, contained 10 statements and scored 3.58 in importance and 3.36 in performance. This cluster addressed the physical and social context in which eating takes place, including atmosphere and setting. The fifth cluster, Production, included seven statements focusing on the origin and characteristics of food. It received average ratings of 3.76 for importance and 3.11 for performance. The sixth cluster, Preparation and Cooking, consisted of eight statements with scores of 3.50 for importance and 3.26 for performance. This cluster addressed perceptions related to ingredient preparation and cooking methods.

The paired t-test results comparing importance and performance across clusters indicated that importance scores were significantly higher than performance scores in five clusters (Production, Preparation and Cooking, Nutrition, Eating Habits, and Food Choice), whereas no significant difference was observed for Eating Environment. Taken together, these findings suggest that participants generally rated importance higher than performance, indicating a gap between awareness and practice. In terms of relative rankings, Nutrition, Food Choice, and Production emerged as the most important clusters, while Eating Environment and Preparation and Cooking showed relatively higher performance ratings.

Of particular note, Food Choice and Nutrition were consistently ranked highest across both dimensions. This pattern may reflect their longstanding prominence in public discourse on healthy eating, making them both highly valued and actionable by consumers. In contrast, Eating Habits, Eating Environment, and Preparation and Cooking scored lower in importance and performance, likely due to their more subjective and individually variable nature. Although our sample included participants from diverse age groups and both sexes, the limited sample size (*n* = 24) prevented meaningful statistical comparisons across these subgroups. To address this limitation, we provide a supplementary pattern-matching graph (Supplementary Figures S1 and S2) that visually illustrates potential directional differences between groups. These results, however, are exploratory in nature and should be interpreted with caution.

### 3.3. Importance and Performance of Healthy Eating Statements

**Figure 3:**
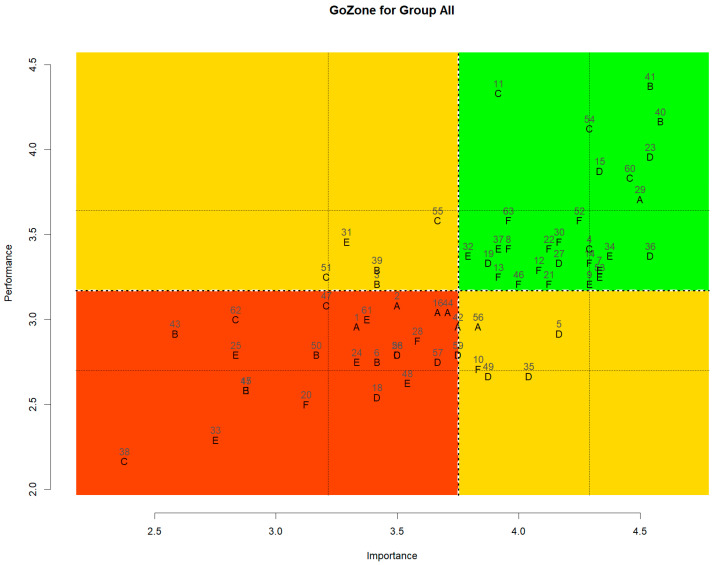
Go-Zone graph for the statements and clusters. 1~63: Statement number, A~F: Clusters.

To gain deeper insights into perceptions of healthy eating, the importance and performance of individual statements were further analyzed and visualized (Figure 3). In concept mapping, this type of graph is referred to as a Go-Zone graph. The graph is divided into four quadrants by lines representing the mean values of the x- and y-axes, where the x-axis indicates importance and the y-axis indicates performance. Also, to provide transparency, the average importance and performance scores for each statement are presented in Supplementary Table S1.

The first quadrant of the Go-Zone graph represents areas where both importance and performance are above average. This quadrant highlights aspects of healthy eating that participants not only consider important but also actively practice. A total of 28 statements were located in this quadrant. These primarily belonged to the Food Choice and Nutrition clusters, including statements such as “*Eat fruit*,” “*Eat less sweet food/Eat less spicy food/Eat less salty food*,” “*Reduce sugar intake*,” “*Reduce salt intake*,” “*Eat plain food*,” “*Eat vegetables*,” “*Choose multigrain rice over white rice*,” “*Eat enough protein*,” “*Eat foods containing vitamins*,” “*Drink enough water*,” “*Eat foods high in fiber*,” and “*Eat a well-balanced diet*.”

These findings suggest that participants show consistently high levels of awareness and performance in food- and nutrient-related domains of healthy eating. As discussed earlier, these two clusters reflect a scientific and functional view of healthy eating, which remains dominant in both personal and institutional understandings. This alignment is reinforced by public nutrition education and health policies. For instance, the General Dietary Guidelines for Koreans [51] emphasize food diversity, reduced intake of sugar, salt, and fat, and adequate water consumption—directly corresponding to items in these clusters. Thus, the prominence of Food Choice and Nutrition reflects a shared understanding of healthy eating at both individual and societal levels. Beyond food and nutrients, this quadrant also included statements from the Eating Environment cluster, such as “*Eat with family and share their emotions*,” “*Eat favorite food*,” “*Eat in a clean environment*,” and “*Eat in a relaxed mood*”. Among them, “*Eat with family and share their emotions*” reflects the cultural value placed on shared meals. Despite the rise of ‘Honbap (meaning eating alone)’ culture in the Republic of Korea [7], participants continue to associate family meals with healthy eating. Prior research supports this view that family meals are associated with improved dietary habits and psychosocial outcomes [52,53]. Hygiene-related statements from the Preparation and Cooking cluster (“*Eat food prepared hygienically*,” “*Prepare food hygienically*,” and “*Eat in a clean environment*”) were also located in this quadrant, indicating high hygiene awareness and performance among participants. This finding suggests that hygienic practices are well integrated into Korean eating behavior and are possibly perceived as baseline expectations. Recent studies have shown a growing emphasis on hygiene in food selection [54,55]. Particularly since the COVID-19 pandemic, public concern regarding food hygiene and safety has increased significantly [56,57,58], potentially reinforcing these behaviors.

The second quadrant of the Go-Zone graph represents behaviors that are performed frequently but are perceived as less important to healthy eating. This quadrant included items such as “*Cook and eat whole food at home*” and “*Check the origin of food*” from the Preparation and Cooking cluster; “*Eat a light breakfast*” from the Eating Habits cluster; and “*Eat at home*” and “*Eat with friends*” from the Eating Environment cluster. These findings suggest that certain behaviors, although commonly practiced, may not be consciously associated with healthy eating by participants.

In particular, “*Eat at home*” reflects the location of eating, while “*Cook and eat whole foods at home*” emphasizes the traditional practice of purchasing raw ingredients and preparing meals entirely at home. Both behaviors remain frequent, but they are no longer perceived as symbolic markers of healthy eating. In other words, home-prepared meals continue to be practiced, yet their centrality in defining what counts as healthy eating has weakened. This represents a different pattern from previous research, such as Mills et al. [59], which highlighted the nutritional and dietary benefits of home cooking.

In addition, the lower importance of “*Eat with friends*” for healthy eating contrasts with the higher importance placed on “*Eat with family and share emotions*,” suggesting that although people may value social eating for its social expectations or relational benefits, they may not see it as supporting personal health. This interpretation aligns with research indicating that social eating can contribute to obesity and negatively affect healthy eating [14].

The third quadrant of the Go-Zone graph contained aspects that consumers neither valued nor performed well in. This area included statements from the Production cluster, such as “*Eat less GMO food*,” “*Eat less processed food*,” “*Eat less instant food*,” “*Eat natural food*,” “*Eat seasonal foods*,” and “*Eat traditional Korean food*.” “*Eat foods rich in unsaturated fatty acids*,” “*Eat low-calorie food*,” and “*Increase calcium intake*” from the Nutrition cluster; “*Avoid delivery food*” from the Eating Habits cluster; “*Choose rice over bread or noodles*,” “*Eat three meals a day*,” and “*Manage one’s diet regularly*” from the Eating Environment cluster.

These findings indicate that attributes once regarded as central to healthy eating—such as reducing processed or instant food consumption, choosing seasonal or natural products, and emphasizing traditional Korean meals—are no longer consistently treated by participants as definitional criteria, marking a departure from conventional understandings of what constitutes healthy eating.

Previous studies have reported that consuming natural foods and limiting processed foods or GMO foods were traditionally perceived as key indicators of a healthy diet [2,5,6,42,60]. In contrast, in this study, these statements were rated low in both importance and performance, suggesting that Korean consumers no longer regard them as central criteria for healthy eating. The low performance further implies that, in practice, consumption of processed and instant foods has increased while intake of natural ingredients has decreased. This shift is closely linked to broader social changes, as the ubiquity, standardization, and convenience of processed and instant foods have expanded alongside busier lifestyles and changing family structures [61]. Although the health implications of processed foods remain debated [62], modern food systems now offer consumers a wide variety of high-quality processed options [63], and recent studies show that consumers no longer classify all processed foods as unhealthy [64]. With regard to GMO foods, European survey data indicated that public concern declined between 2010 and 2019 [65], which is consistent with our findings.

At the same time, the statements “*Avoid delivery foods*” and “*Avoid eating out*” were also located in this quadrant. Unlike “*Cook and eat whole food at home*” or “*Eat at home*” in Quadrant 2, which reflect a location-based practice that remains frequent, these items represent behaviors aimed at deliberately avoiding meals prepared outside the home. Their low importance and low performance scores indicate that participants neither frequently practiced these behaviors nor regarded them as central to healthy eating. Traditionally, such avoidance was linked to the belief that home-prepared meals were inherently safe and healthy [66]. However, in today’s busy society, eating out or ordering food deliveries has moved beyond being a mere option and has become an important aspect of dietary practices [58,67]. In particular, since the COVID-19 outbreak, the frequency of home meal deliveries has increased significantly [20,58], leading to a shift in the concept of meals from a simple location-based distinction (in-home vs. out-of-home) to a more complex and multifaceted concept.

Such perceptual and dietary changes are not unique to the Republic of Korea. They have also been observed in other cultures where household consumption of takeout and processed foods has significantly increased [68]. Moreover, studies have reported that people consider their mood more important than before when making food choices, and eating less convenient foods is not necessarily nutritionally advantageous [69].

These findings contradict earlier studies that align with the prevailing conventional view—that reducing away-from-home food expenditure can improve dietary quality [70] and that ultra-processed or convenience foods are generally associated with poorer nutritional quality [71,72]. However, this discrepancy can be explained by the fact that the present study approached the issue from a different analytical dimension, focusing not on the nutritional composition itself but on a perceptual shift: the recognition that such attributes are no longer consistently regarded as central markers when defining what constitutes healthy eating.

A similar de-centering is observed for ‘hansik (traditional Korean food)’. Although the traditional Korean diet has long been perceived as nutritionally superior and health-promoting [73], awareness has expanded that commonly consumed dishes—kimchi and soups/stews, in particular—can be major contributors to dietary sodium [74,75]. In parallel, the health benefits of evidence-based patterns such as the Mediterranean diet have been widely disseminated [76], indicating a shift in the information environment for “healthy eating” beyond a hanik-only frame toward a more diversified set of criteria.

Lifestyle-related statements also illustrate this perceptual shift. “*Avoid eating while watching TV or using a mobile phone*” scored low in both importance and performance, suggesting that participants do not associate media use while eating with healthy eating, even though such behaviors are frequent in daily life. This pattern may reflect lifestyle changes during COVID-19, when sedentary behaviors increased and digital device use for education and work became more pervasive, further reinforcing such habits [77]. Moreover, this suggests that behaviors previously regarded as unhealthy habits are no longer consistently perceived as related to healthy eating, reflecting a broader shift in perceptions alongside lifestyle changes. In fact, several studies have reported increased TV viewing during mealtimes after COVID-19 [78,79,80], providing evidence that people’s perceptions regarding non-food elements associated with their eating habits and lifestyle patterns may have changed.

The fourth quadrant of the Go-Zone graph represents behaviors that participants perceive as highly important but do not frequently practice. This quadrant included statements such as “*Eat adequate calories to maintain a healthy weight*,” “*Supplement nutrients as needed by age*,” “*Eat low-fat food*,” and “*Reduce carbohydrates intake*” from the Nutrition cluster; “*Cook with less oil*” from the Food Choice cluster; and “*Eat organic food*” from the Production cluster.

These findings suggest a notable gap between the perceived importance of weight management and actual dietary behavior. For instance, statements like “*Eat adequate calories to maintain a healthy weight*,” “*Eat low-fat food*,” and “*Reduce carbohydrates intake*” indicate that participants are highly aware of the nutritional strategies associated with weight control. This aligns with previous studies that have emphasized the significance of calorie regulation and fat intake in managing body weight [81,82,83]. Despite this awareness, performance scores remained relatively low, pointing to challenges in translating knowledge into consistent practice. One possible explanation is that while individuals recognize the health benefits of maintaining a healthy weight, actual dietary regulation—such as portion control, macronutrient balancing, and low-fat cooking—may be constrained by taste preferences, cooking habits, or lack of nutritional planning. Moreover, the inclusion of “*Eat organic food*” in this quadrant suggests that while participants acknowledge the health or environmental benefits associated with organic consumption, there are factors that may hinder consistent practice. This discrepancy between intention and behavior highlights the importance of targeted education and structural interventions to reduce the barriers to practicing health-promoting behaviors.

Through the Go-Zone graph analysis in this study, we found that perceptions of healthy eating are changing in various ways. Participants rated the importance and practice levels of nutritional management and hygienic cooking and consumption as high but showed variation across other areas—for example, while frequently eating home-cooked meals, eating with friends, and following traditional eating habits, they were less aware of these behaviors’ connections to health or rated their importance and practice levels as low. In particular, following the COVID-19 pandemic, interest in hygiene has increased significantly, and the use of delivery food and home meal replacements (HMR) has become widespread, making the traditional notion that “Eating at home is healthy” no longer consistently associated with health.

Additionally, the traditional dichotomy between home-cooked meals (meals prepared at home) and dining out (meals prepared outside the home) has become more complex and flexible, evolving into categories such as home-cooked meals with dining-out characteristics, dining-out meals with home-cooked characteristics, and other hybrid forms [84]. The emergence of various meal types—such as home-style restaurants, HMR products with dining-out concepts, and HMR products designed to reflect demand for home-cooked meals—has blurred the boundaries between eating out and eating at home, influencing fixed notions of healthy eating. These results suggest that perceptions of healthy eating are being reshaped not only by nutritional and scientific knowledge but also by sociocultural contexts and environmental changes. Therefore, to promote healthy eating behaviors, customized education and policy approaches that take into account these evolving perceptions and lifestyles are necessary.

## 4. Conclusions

### 4.1. Theoretical Contributions and Practical Implications

In this study, concept mapping was used to (1) investigate the healthy eating perceptions of Koreans aged 16–55 years and (2) identify the key components of healthy eating as perceived by this subpopulation within the context of the current socioeconomic environment and food culture. As such, this study provides an in-depth analysis of contemporary perceptions of healthy eating among Koreans aged 16–55 years, revealing that their understanding of healthy eating extends beyond nutrients and food intake to encompass a diverse range of eating-related aspects.

Korean consumers articulated their perceptions of healthy eating across six thematic categories: Food Choice, Nutrition, Eating Habits, Preparation and Cooking, Eating Environment, and Production. These perceptions are shaped by Korean food culture and the evolving food consumption environment. Contextual factors such as time of day, atmosphere, emotions, companionship, and considerations of hygiene and safety are among the elements that Koreans perceive to contribute to healthy eating in this study. We also found significant changes in perceptions of practices traditionally considered healthy, such as eating home-cooked meals instead of dining out, using natural and unprocessed ingredients, and consuming Korean food. The significance of this study lies in uncovering Koreans’ perceptions of healthy eating in light of current trends and socio-cultural perspectives, thereby offering a multifaceted perspective on what constitutes healthy eating.

### 4.2. Study Limitations and Future Research Directions

This study has several limitations. First, the small sample size (n = 24) limits the generalizability of the findings. Although participants were drawn from diverse age groups (16–55) and both sexes, the small sample size made it difficult to conduct meaningful subgroup comparisons. Accordingly, formal statistical tests such as t-tests or ANOVA could not be performed, and subgroup comparisons remained at an exploratory level. To illustrate potential group-level trends, a supplementary pattern-matching graph (Supplementary Figure S1) was presented, and the average importance and performance scores for all 63 statements were provided in Supplementary Table S1 to enable readers to examine the values underlying the Go-Zone graph. However, these results cannot be statistically confirmed or generalized; therefore, future studies should recruit a sufficient number of participants to enhance the validity and generalizability of the findings. In addition, during the statement development process, although the content validity of the statements was established through expert review and consensus, reliability testing of the tool (such as test–retest or consistency across expert ratings) was not conducted. Future research should address this by incorporating formal reliability analyses to further strengthen the robustness of the instrument.

Second, the sample was restricted to participants from Seoul with relatively high socioeconomic status, which limits the generalizability of the findings. Because perceptions of healthy eating are strongly influenced by socioeconomic status [8,85,86], different results may emerge when participants are drawn from other socioeconomic backgrounds. Future studies should recruit participants from diverse regions and socioeconomic contexts across the Republic of Korea.

Third, perceptions of healthy eating may vary depending on an individual’s physical health status. Not only body measurements such as weight and body fat, but also medical conditions such as obesity, hypertension, and diabetes may influence these perceptions. Thus, future research comparing perceptions across various health conditions could contribute to the development of more specific strategies for healthy eating.

Fourth, since this study examined perceptions among general consumers rather than professionals, it was not possible to determine whether their perceptions of healthy eating were actually “healthy.” Future research should involve experts to compare existing consumer perceptions with evidence-based food and nutrition perspectives to provide more specific methods and strategies for healthy eating. Longitudinal studies could also track how perceptions change in response to evolving dietary environments, while comparative studies across regions and socioeconomic groups could help identify contextual variations in healthy eating perceptions. Furthermore, these insights could be translated into tailored education programs for different population groups, offering practical guidance to promote healthier eating practices.

Finally, the findings regarding home cooking—where such practices remain frequent but are no longer perceived as central markers of healthy eating—highlight a nuanced and somewhat contradictory pattern. While home cooking continues to be practiced regularly, its declining centrality suggests that traditional assumptions about its role in healthy eating may not fully align with contemporary perceptions. These findings should therefore be interpreted within the broader context of changing dietary environments, recognizing that evolving food cultures and lifestyles may reshape how people define and value healthy eating.

In today’s rapidly changing dietary environment, healthy eating is increasingly defined by diverse criteria, and individuals make food choices based on varying interpretations of what constitutes healthy eating. These changes extend beyond the academic sphere, offering important implications for policymakers, health educators, and the food industry. Based on the findings of this study, the food industry, in particular, is encouraged to conduct continued research on healthy eating and to provide practical and culturally appropriate approaches for promoting healthy eating within the contemporary socio-cultural context.

## Figures and Tables

**Table 1:** Differences between importance and performance by the clusters.

Cluster	Importance Mean ± SD	Performance Mean ± SD	t
Food choice	3.96 ± 0.51	3.22 ± 0.55	5.490 *** ^(1)^
Nutrition	3.99 ± 0.50	3.08 ± 0.33	7.036 ***
Eating habits	3.62 ± 0.51	3.03 ± 0.56	4.390 ***
Eating environment	3.58 ± 0.57	3.36 ± 0.36	1.745 ^NS (2)^
Production	3.76 ± 0.73	3.11 ± 0.74	4.609 ***
Preparation and cooking	3.50 ± 0.43	3.26 ± 0.51	2.354 *

(1) * *p* < 0.05, *** *p* < 0.001. (2) ^NS^: Not significant.

## Data Availability

The data presented in this study are available on request from the corresponding author.

## Supplementary Materials

The following supporting information can be downloaded at: https://www.mdpi.com/article/10.3390/nu17182941/s1, Figure S1: Pattern matching graph comparing the mean importance and performance ratings of healthy eating perceptions across age groups; Figure S2: Pattern matching graph comparing the mean importance and performance ratings of healthy eating perceptions across sex groups; Table S1: Average importance and performance scores for the 63 healthy eating statements.
