# Supplementary material for: Perceptions Regarding Healthy Eating Based on Concept Mapping
Source: Nutrients. 2025 Sep 12;17(18):2941. doi: 10.3390/nu17182941 (PMC12472633; doi:10.3390/nu17182941)
Supplement: Supplementary file 1 [file nutrients-17-02941-s001.zip › nutrients-3845625-supplementary.pdf]

**Supplementary Figure S1**. Pattern matching graph comparing the mean importance and performance ratings of healthy eating perceptions across age groups.

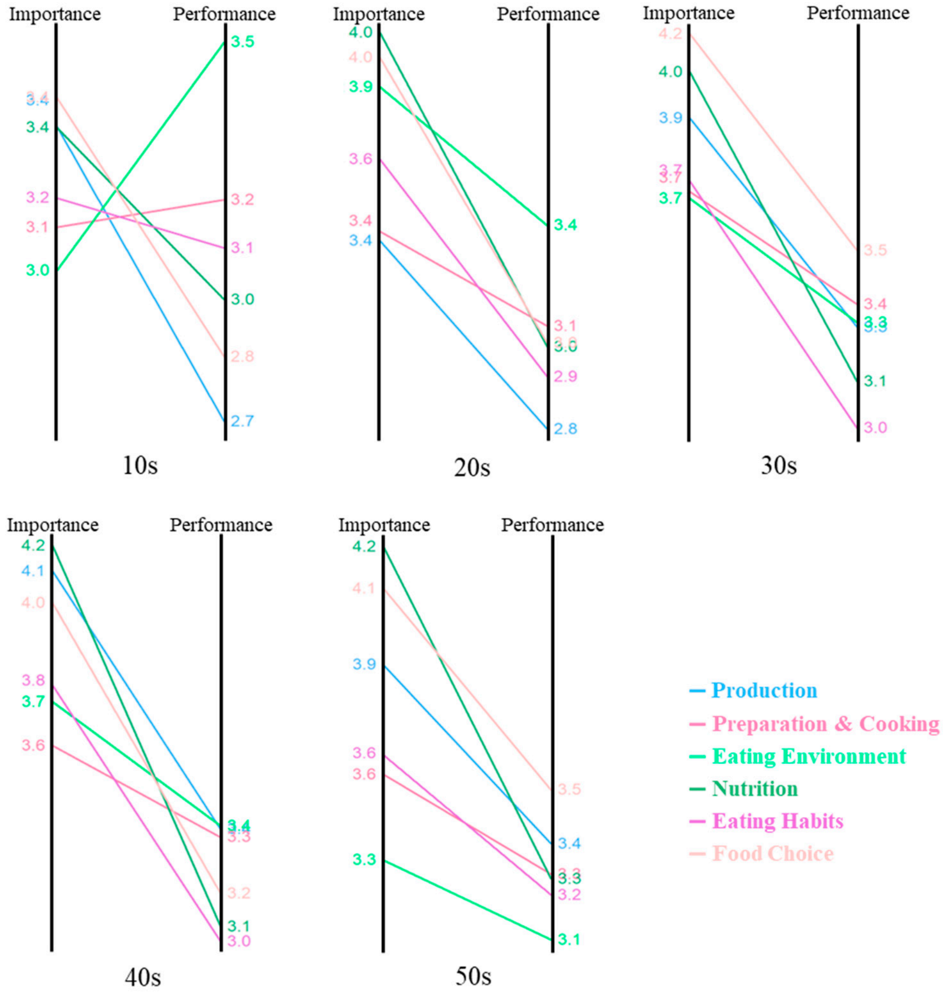

**Supplementary Figure S2.** Pattern matching graph comparing the mean importance and performance ratings of healthy eating perceptions across sex groups.

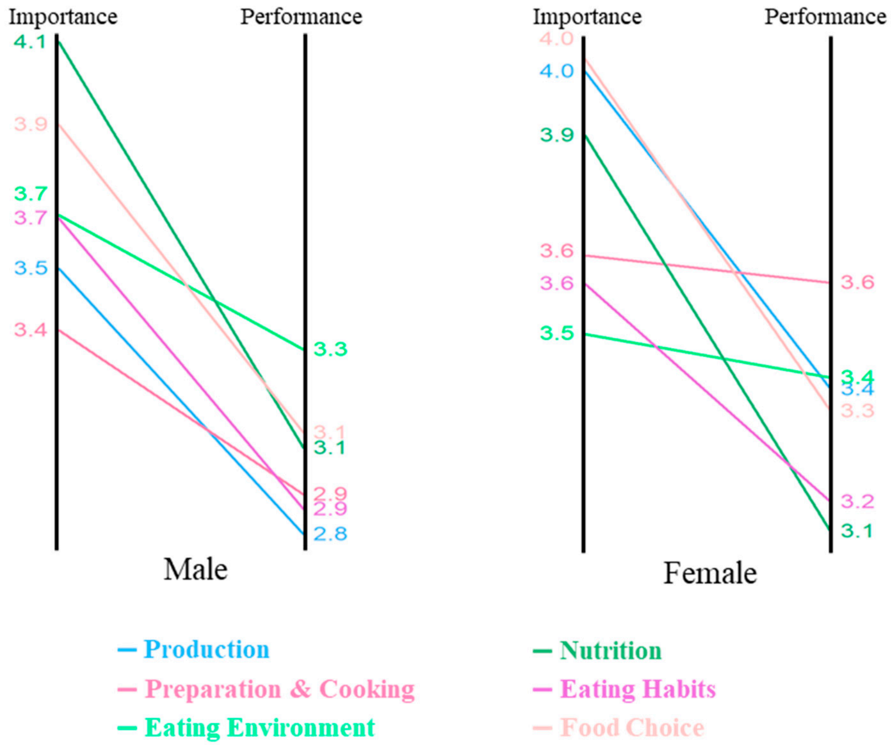

**Supplementary Table S1.** Average importance and performance scores for the 63 healthy eating statements.

| Cluster | Statements€ | Importance | Performance |
|---|---|---|---|
| | | Mean ± SD | Mean ± SD |
| **Production** | 1. Eat less GMO foods | 3.33±1.27 | 2.96±1.40 |
| | 2. Eat less processed food | 3.5±1.14 | 3.08±1.18 |
| | 16. Eat less instant food | 3.67±1.01 | 3.04±1.08 |
| | 29. Eat fresh food | 4.50±0.59 | 3.71±1.04 |
| | 42. Eat natural food | 3.75±1.03 | 2.96±1.04 |
| | 44. Eat seasonal food | 3.71±1.04 | 3.04±1.04 |
| | 56. Eat organic food | 3.83±0.92 | 2.96±1.00 |
| **Preparation and Cooking** | 3. Cook and eat whole food at home | 3.42±1.06 | 3.21±1.14 |
| | 6. Buy healthy food | 3.42±0.88 | 2.75±0.94 |
| | 39. Check the origin of food | 3.42±1.10 | 3.29±1.40 |
| | 40. Eat food prepared hygienically | 4.58±0.5 | 4.17±0.82 |
| | 41. Prepare food hygienically | 4.54±0.59 | 4.38±0.77 |
| | 43. Cook with processed food | 2.58±1.02 | 2.92±1.1 |
| | 45. Cook less and eat natural food | 2.88±1.15 | 2.58±1.14 |
| | 50. Cook your own meals | 3.17±1.13 | 2.79±1.1 |
| **Eating Environment** | 4. Eat with family and share their emotion | 4.29±0.91 | 3.42±1.21 |
| | 11. Eat favorite food | 3.92±1.32 | 4.33±0.70 |
| | 26. Avoid eating while watching TV or using a mobile phone | 3.5±1.53 | 2.79±1.28 |
| | 38. Avoid eating out | 2.38±0.97 | 2.17±1.09 |
| | 47. Eat in a quiet atmosphere | 3.21±1.25 | 3.08±1.02 |
| | 51. Eat at home | 3.21±1.14 | 3.25±1.07 |
| | 54. Eat in a clean environment | 4.29±0.75 | 4.12±0.90 |
| | 55. Eat with friends | 3.67±1.09 | 3.58±1.14 |
| | 60. Eat in a relaxed mood | 4.46±0.78 | 3.83±1.05 |
| | 62. Eat alone | 2.83±1.40 | 3.00±1.18 |
| **Nutrition** | 5. Eat adequate calories to maintain a healthy weight | 4.17±0.64 | 2.92±0.83 |
| | 15. Eat enough protein | 4.33±0.64 | 3.88±0.85 |
| | 18. Eat foods with unsaturated fatty acids | 3.42±0.83 | 2.54±0.88 |
| | 19. Eat foods containing vitamins | 3.88±0.90 | 3.33±1.05 |
| | 23. Drink enough water | 4.54±0.66 | 3.96±0.75 |
| | 27. Eat foods high in fiber | 4.17±0.76 | 3.33±1.17 |
| | 35. Supplement nutrients as needed by age | 4.04±1.04 | 2.67±1.13 |
| | 36. Eat a well-balanced diet | 4.54±0.66 | 3.38±1.01 |
| | 49. Eat low-fat food | 3.88±0.99 | 2.67±0.76 |
| | 57. Eat low-calorie food | 3.67±0.96 | 2.75±0.79 |
| | 58. Increase calcium intake | 3.5±0.88 | 2.79±0.88 |
| | 59. Reduce carbohydrates intake | 3.75±1.15 | 2.79±1.06 |

| | | | |
|---|---|---|---|
| Eating Habits | 7. Do not overeat | 4.33±0.70 | 3.29±1.16 |
| | 9. Eat at regular times | 4.29±1.00 | 3.21±1.22 |
| | 17. Avoid delivery food | 2.88±1.26 | 2.58±0.35 |
| | 24. Choose rice over bread or noodles | 3.33±1.27 | 2.75±1.15 |
| | 25. Eat traditional Korean food | 2.83±1.13 | 2.79±1.06 |
| | 31. Eat a light breakfast | 3.29±0.95 | 3.46±1.32 |
| | 32. Eat breakfast | 3.79±1.25 | 3.38±1.44 |
| | 33. Eat a traditional Korean breakfast | 2.75±1.11 | 2.29±1.23 |
| | 34. Avoid late-night eating | 4.38±0.88 | 3.38±1.41 |
| | 37. Read nutrition labels | 3.92±1.06 | 3.42±1.47 |
| | 48. Manage one's diet regularly | 3.54±1.14 | 2.62±1.21 |
| | 53. Eat slowly | 4.33±0.70 | 3.25±1.11 |
| | 61. Eat 3 meals a day | 3.38±1.31 | 3.00±1.22 |
| Food Choice | 8. Eat fruit | 3.96±0.91 | 3.42±1.10 |
| | 10. Cook with less oil | 3.83±0.87 | 2.71±1.08 |
| | 12. Eat less sweet food | 4.08±0.83 | 3.29±0.86 |
| | 13. Eat less spicy food | 3.92±0.97 | 3.25±1.15 |
| | 14. Eat less salty food | 4.29±0.81 | 3.33±1.13 |
| | 20. Use sugar or salt substitutes | 3.12±1.12 | 2.50±1.14 |
| | 21. Reduce sugar intake | 4.12±0.90 | 3.21±0.98 |
| | 22. Reduce salt intake | 4.12±0.8 | 3.42±0.93 |
| | 28. Avoid food additives | 3.58±1.21 | 2.88±1.19 |
| | 30. Eat plain food | 4.17±0.76 | 3.46±1.32 |
| | 46. Use less seasoning | 4.00±0.88 | 3.21±1.14 |
| | 52. Eat vegetables | 4.25±0.85 | 3.58±0.97 |
| | 63. Choose multigrain rice over white rice | 3.96±1.12 | 3.58±1.10 |
